# The Inflammation-Based Glasgow Prognostic Score as a Prognostic Factor in Patients with Intensive Cardiovascular Care Unit

**DOI:** 10.3390/medicina55050139

**Published:** 2019-05-15

**Authors:** Servet Altay, Muhammet Gürdoğan, Muhammed Keskin, Fatih Kardaş, Burcu Çakır

**Affiliations:** 1 Department of Cardiology, School of Medicine, Trakya University, 22030 Edirne, Turkey; drmgurdogan@gmail.com (M.G.); drfatihkardas@gmail.com (F.K.); burcu_cakir2@hotmail.com (B.Ç.); 2 Department of Cardiology Health Sciences University, Sultan Abdulhamid Han Training and Research Hospital, 34010 Istanbul, Turkey; drmuhammedkeskin@gmail.com

**Keywords:** Glasgow Prognostic Score, albumin, C-reactive protein, inflammation, mortality

## Abstract

*Background:* The Glasgow prognostic score (GPS), which is obtained from a combination of C-reactive protein (CRP) and serum albumin level, predicts poor prognoses in many cancer types. Systemic inflammation also plays an important role in pathogenesis of cardiovascular diseases. In this study, we aimed to investigate the effect of inflammation-based GPS on in-hospital and long-term outcomes in patients hospitalized in intensive cardiovascular care unit (ICCU). *Methods:* A total of 1004 consecutive patients admitted to ICCU were included in the study, and patients were divided into three groups based on albumin and CRP values as GPS 0, 1, and 2. Patients’ demographic, clinic, and laboratory findings were recorded. In-hospital and one-year mortality rates were compared between groups. *Results:* Mortality occurred in 109 (10.8%) patients in in-hospital period, 82 (8.1%) patients during follow-up period, and thus, cumulative mortality occurred in 191 (19.0%) patients. Patients with a high GPS score had a higher rate of comorbidities and represented increased inflammatory evidence. In the multivariate regression model there was independent association with in-hospital mortality in GPS 1 patients compared to GPS 0 patients (Odds ratio, (OR); 5.52, 95% CI: 1.2–16.91, *p* = 0.025) and in GPS 2 patients compared to GPS 0 patients (OR; 7.01, 95% CI: 1.39–35.15, *p* = 0.018). A higher GPS score was also associated with a prolonged ICCU and hospital stay, and increased re-hospitalization in the follow-up period. *Conclusion:* Inflammation based GPS is a practical tool in the prediction of worse prognosis both in in-hospital and one-year follow-up periods in ICCU patients.

## 1. Introduction

Cardiovascular diseases are the leading cause of death worldwide and there are an increasing incidence of them [1]. Intensive cardiovascular care units (ICCU) are the units in which patients with the leading causes of death, especially acute coronary syndrome (ACS), acute decompensated heart failure (AHF), and malignant arrhythmia are followed-up. Despite advancements in medical and interventional treatments, in-hospital and one-year follow-up mortality rates of ACS and AHF remain high [2,3] Therefore, it is crucial to identify high-risk patients hospitalized in ICCU, particularly those with ACS or AHF.

Recently, many studies have shown that inflammation plays an important role in etiopathogenesis of cardiovascular diseases. Inflammatory markers have been shown to have an important role in pathogenesis and prognosis of coronary artery disease (CAD), ACS, AHF, and cardiac arrhythmias [4,5,6,7]. Similarly, albumin based nutritional indices have been associated with mortality and prognosis in ACS patients [8]. Inflammation is known to play a role in pathogenesis of cancer, which is the second most common cause of mortality [9,10]. Studies have demonstrated that the Glasgow prognostic score (GPS), which is a simple and useful score and obtained using C-reactive protein (CRP) and albumin values is a practical tool in the determination of prognosis in many cancer types. In this study, we aimed to investigate the effect of GPS on in-hospital and one-year prognosis in patients followed in ICCU where inflammatory and nutritional status are important and mortality rates are high.

## 2. Material and Methods

### 2.1. Subject Selection

The study was conducted in Trakya University Medical Faculty, which has the highest patient volume in Trakya region of Turkey. The study was designed as a retrospective and cross-sectional study, and all patients hospitalized in ICCU between 1 December 2016 and 1 December 2017 were included in the study. Inclusion and exclusion criteria and number of patients are shown in Figure S1. The number of patients hospitalized in ICCU during the study period was 1097. Patients without albumin and/or CRP values (that are needed to calculate GPS score) were excluded, leaving 1004 patients, 657 men and 347 women, in the study population. The age range of the subjects was 18–94 and the mean age was 66 years. Files of all patients were examined in detail and demographic, clinic, and laboratory parameters were recorded. In-hospital mortality and prognosis data of the patients were obtained from the file records. As our hospital is a regional hospital, the majority of patients attended follow-up visits. As such, 12-month follow-up data of the patients were obtained from hospital records and telephone interviews. Survival status was confirmed from the nationwide Identity Participation System in a few patients (5%) who could not be reached. Study protocol was approved by Trakya University Scientific Research Ethics Committee with TUTF-BAEK 2019/10 numbered decision.

### 2.2. Definitions

The GPS score was calculated as patients with an increased CRP level (>1.0 mg/dL) and low albumin level (<3.5 g/dL) were assigned a GPS of 2. Patients with only one of these biochemical abnormalities were allocated a GPS of 1. Patients with neither of these abnormalities were assigned a GPS of 0.

The Glomerular filtration rate (GFR) (in mL/min per 1.73 m^2^) was estimated using the abbreviated equation developed by the Modification of Diet in Renal Disease (MDRD) Formula [11]. Body mass index (BMI) was calculated as weight in kilograms divided by height in meters square (kg/m^2^).

We defined AHF on the basis of the diagnostic criteria of the European Society of Cardiology Heart Failure Guidelines [3]. ACS was defined as the presence of unstable angina pectoris, non-ST-elevation, or ST-elevation myocardial infarction [12]. Dyslipidemia was defined on the basis of a prior diagnosis of dyslipidemia presence of an abnormal lipid profile or current treatment with antidyslipidemic medication. Hypertension was defined as a blood pressure ≥140 mmHg and/or ≥90 mmHg, and/or use of antihypertensive agents. Diabetes mellitus (DM) was diagnosed with the criteria of the American Diabetes Association (ADA) [13]. CAD was diagnosed by a history of myocardial infarction and coronary intervention, by angiographic greater than 50% stenosis of at least one major coronary vessel. Stroke was defined as affected impairment of brain function, which resulted in an inability limb movement or understanding or formulating speech.

### 2.3. Statistical Examinations

The Kolmogorov-Smirnov test was used for testing normality. Continuous variables with normal distribution were expressed as mean ± SD and compared using one-way analysis of variance. Then, the Tukey post-hoc test was performed to reveal the statistical difference between the groups. Continuous variables with skewed distribution were expressed as median and interquartile range and compared using the Kruskal-Wallis test and Bonferoni-corrected Mann-Whitney U test was performed to reveal the statistical difference between the groups. Categorical variables were expressed as numbers and percentages and Pearson’s chi-square or Fisher’s exact tests were used to evaluate the differences. Hierarchical logistic regression analysis was used to evaluate the univariable and multivariable confounders. Multivariate analysis using stepwise logistic regression models (backward elimination) tested variables that were significant at *p* < 0.05 in the univariate analysis. The odds ratio (OR) indicates the relative risk of death of groups. In multivariable models, confounders in multivariate analysis as predictors of in-hospital and long-term mortality were considered. The median survival times of three groups were compared using the Kaplan-Meier survival method. Differences between the groups were analyzed by the log-rank test. A two-tailed *p* value of <0.05 was considered as statistically significant and 95% CIs were presented for all odds ratios and hazard ratios. Data were analyzed by Statistical Package for Social Sciences (SPSS) version 20.0 for Windows (IBM, Armonk, New York, NY, USA).

## 3. Results

A total of 1004 patients (mean age, 65.4 ± 13.7; 657 men {65.4 %}) who followed in our intensive cardiovascular care with variable indications were included in the study. Of these patients, 109 (10.8%) died during hospitalization and 82 (8.1%) died during the follow-up period (median follow-up time, 9.1 months). Of these patients, 536 (53.3%) had a GPS of 0, 391 (38.9%) had a GPS of 1, and 77 (7.6%) had a GPS of 3 on admission.

Table S1 shows the baseline characteristics according to the GPS. Patients with a higher GPS score were older, had higher incidences of DM, heart failure, atrial fibrillation (AF), chronic kidney disease (CKD), and chronic pulmonary disease, and were smoking less compared to those with a lower GPS score. Left ventricular ejection fraction (LVEF) was significantly lower in the high GPS group. Among admission laboratory parameters, leukocyte, neutrophil, TG, creatinine, blood glucose, N-terminal brain natriuretic peptide (NT-ProBNP), total bilirubin, aspartate aminotransferase (AST), and alanine aminotransferase (ALT) values were higher, while hemoglobin, GFR, and total/LDL cholesterol levels were significantly lower in the high GPS group. Evaluating medical therapies in the ICCU, the use of aspirin, P2Y12 inhibitors, and statins was more common in the low GPS group, while diuretics, inotropic agents, total parenteral nutrition (TPN), and antimicrobial agents were more commonly used in the high GPS group. ICCU and total hospitalization durations were significantly longer in the high GPS group (Table S1).

The majority of ICCU patients were hospitalized due to the diagnosis of ACS (65.3%) and AHF (12.9%), with the diagnosis of ACS more common in the low GPS group, and the diagnosis of AHF more common in the high GPS group. No significant difference was found between the groups in terms of the other diagnoses (Table S2).

During the hospitalization, the incidences of cardiopulmonary arrest (CPA) and acute kidney failure were significantly higher in the high GPS group (*p* < 0.001) (Table S3). The in-hospital mortality gradually increased with an increase in GPS scores (GPS = 0 vs. GPS = 1 vs. GPS = 2: 4.1% vs. 16.4% vs. 29.9%, *p* < 0.001). The cumulative mortality was consistently more significant in patients with a higher GPS than in those with a lower GPS in both genders. This finding was also consistent when compared to under and above patients aged 60 years. The rates of mortality and the number of the days of hospitalization were also significantly higher in the high GPS group (Table S3, Figure S2).

In the univariate regression analysis, GPS (1 vs. 0), GPS (2 vs. 0), age, heart failure, CKD, presence of AF, absence of hypertension, elevated levels of leucocyte count, creatinine, total bilirubin, NT-ProBNP, ALT and AST, and low levels of hemoglobin, total cholesterol, and lower LVEF were found as the independent predictors of in-hospital mortality (Table S4). In the multivariate regression model, only GPS and NT-ProBNP values were found as the predictors of in-hospital mortality. In the multivariate regression model there was a significantly increased association with in-hospital mortality in GPS 1 patients compared to GPS 0 patients (OR; 5.52, 95% CI: 1.2–16.91, *p* = 0.025) and in GPS 2 patients compared to GPS 0 patients (OR; 7.01, 95% CI: 1.39–35.15, *p* = 0.018).

## 4. Discussion

The main finding of our study was that high GPS scores were associated with an increased in-hospital mortality and total follow-up mortality. In the multivariate analysis, a high GPS score was found to be the significant dominant factor in in-hospital mortality. Studies have reported the importance of inflammation in pathogenesis of cardiovascular diseases. It plays an important role in pathogenesis of numerous cardiovascular diseases from endothelial damage to atherosclerotic plaque formation and plaque rupture in CAD and ACS, disease progression in heart failure, and the development of arrhythmias, especially atrial fibrillation [4,5,6,7]. Concordantly, studies have shown the association of inflammatory markers especially CRP which is among the GPS score variables, interleukin-6 (IL-6), tumor necrosis factor alpha, pentraxin 3 and neutrophil-to-lymphocyte ratio with poor prognosis in cardiovascular diseases [5,14,15,16]. Inflammation has been shown to play role in the growth, invasion, and metastasis of tumors in many cancer types [9,10]. Inflammatory markers such as CRP, albumin, neutrophil, and lymphocyte count have been associated with mortality in numerous cancer types [17,18]. From this point of view, cancer shows similarity with cardiovascular diseases. Even there are analyses suggesting that heart failure and cancer may have a common etiologic basis depending on the proinflammatory state [19]. Our study indicates that GPS score based on serum CRP and albumin levels may show inflammatory status; it might also have prognostic value in ICCU patients as in cancer patients. In our study, elevated inflammatory markers such as leukocyte and neutrophil counts in the high GPS group support this result.

Albumin is one of the variables in the GPS score and maintains plasma oncotic pressure, as well as antioxidant and anti-inflammatory features by carrying inflammatory substances [20,21]. Therefore, lower levels of albumin may cause increased inflammation and oxidative stress. Additionally, IL-6 which is released in the case of inflammation increases the production of acute phase reactants in liver and downregulates the production of albumin [22]. Thus, lower levels of albumin are considered a marker of inflammation.

In addition, a low albumin level is an indicator of malnutrition and has been associated with higher mortality in cancer and ACS [8,23]. Furthermore, hypoalbuminemia was found to be associated with unfavorable in-hospital prognosis and long-term mortality [24,25]. This effect of low albumin in AHF patients has been attributed to general systemic dysfunction rather than cardiac performance. In addition, hypoalbuminemia is associated with coagulation factors and prothrombotic status [26]. In the light above mentioned statements, low levels of albumin could be associated with poor prognosis in ICCU patients.

GPS is a combination of CRP and albumin and is an exclusive index of systemic inflammation and malnutrition. GPS has been determined as a predictor of mortality, especially in several cancer types including hepatocellular, cervix and non-small cell lung cancer (NSCLC), and diffuse large cell lymphoma [27,28,29,30]. This score has also been associated with higher mortality in acute worsening of pulmonary fibrosis [31]. Previous studies and our study revealed that the GPS score may identify a patient with poor condition in many diseases rather than indicating any specific disease or clinical outcome. In the present study, comorbidities such as DM, CKD, AF, and HF had admission due to AHF with a poor prognosis. Further, laboratory findings associated with negative prognosis such as NT-proBNP, creatinine, AST and ALT enzymes, and treatment with negative outcomes such as inotropic agents, antimicrobial therapy and TPN were higher in the high GPS group [1,2,3]. In addition, low levels of hemoglobin, GFR, and LVEF associated with negative outcomes were also more common in the high GPS group. These results show that GPS score may remain high in patients with a poor prognosis in many diseases.

Another important finding of our study was that high GPS scores were associated with prolonged ICCU and hospital stay and re-hospitalization during the follow-up period. Previous studies have mainly investigated the relationship between GPS score and mortality [27,28,29,30]. There is only one study in the literature investigating the relationship between GPS score and duration of hospitalization. In that study, higher GPS scores were found to be associated with prolonged intensive care unit stay following pneumonectomy in patients with NSCLC [32]. Despite different patient populations and different indication of hospitalization, the results of our study were in line with that study. Our study demonstrated that higher GPS scores were associated with longer ICCU and total hospital stay in patients followed in ICCU with variable indications. In addition, our study showed that the rate of rehospitalization during follow-up was higher in the patients with a high GPS score. More frequent outpatient clinic controls might be considered in these patients after discharge.

GPS score has been usually studied in cancer patients, and studies including cardiovascular diseases are limited. Further, there is no study in the literature including all ICCU patients. To the best of our knowledge, our study is the first conducted the patients hospitalized in ICCU. The current study showed that the GPS score might be a simple and practical score predicted both in-hospital and long-term mortality.

### Limitations

The most important limitation of this study was its retrospective design and being conducted in a single center. Retrospective nature of the study might have caused some factors that affected outcomes to be overlooked. Our study will pioneer further multicenter, prospective studies with long follow-up duration, investigating the use of GPS in prediction of prognosis in ICCU patients.

## 5. Conclusions

High GPS scores are an independent predictor of in-hospital and short-term follow-up mortality in ICCU patients. GPS scores determined using albumin and CRP levels are a simple and practical index in prediction of prognosis in patients hospitalized in ICCU.

## Supplementary Materials

The following are available online at https://www.mdpi.com/1010-660X/55/5/139/s1. Figure S1. Inclusion and exclusion criteria. Figure S2. Kaplan-Meier survival analysis. Table S1. Relationships between clinical characteristics and the GPS in patients followed in intensive care unit. Table S2. The distribution of diagnosis of ICCU patients on admission. Table S3. Relationships between clinical events and the GPS in patients followed in intensive care unit and follow-up period. Table S4. Logistic regression of in-hospital mortality for patients followed in intensive care unit.
