# Supplementary material for: The Inflammation-Based Glasgow Prognostic Score as a Prognostic Factor in Patients with Intensive Cardiovascular Care Unit
Source: Medicina (Kaunas). 2019 May 15;55(5):139. doi: 10.3390/medicina55050139 (PMC6572028; doi:10.3390/medicina55050139)
Supplement: Supplementary file 1 [file medicina-55-00139-s001.pdf]

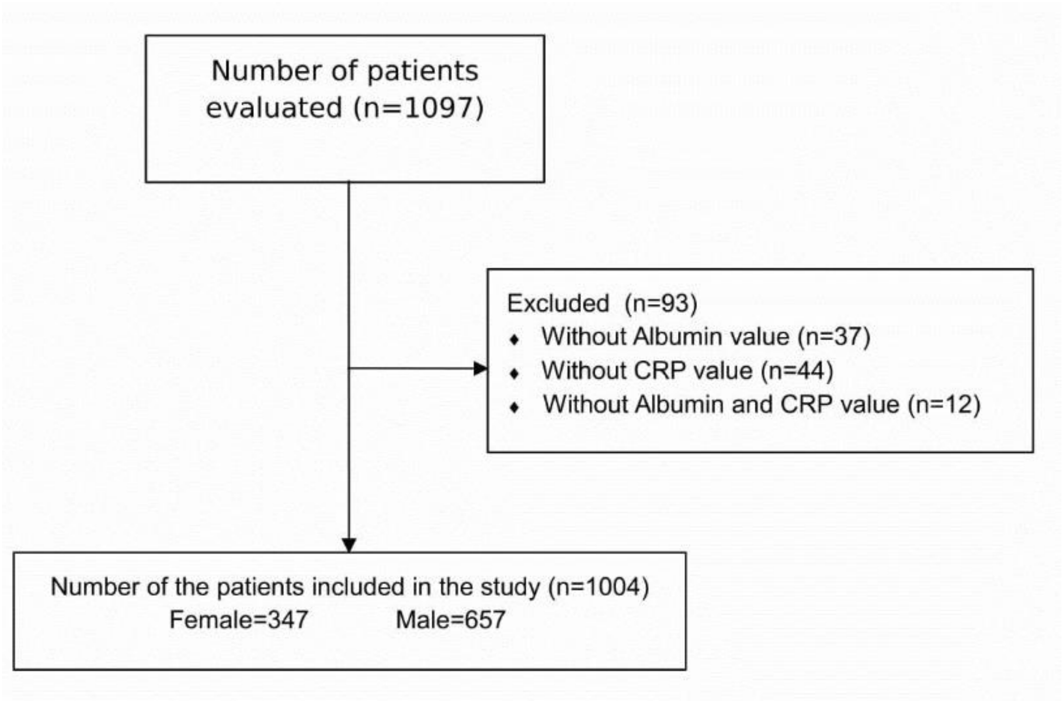

Figure S1. Inclusion and exclusion criteria.

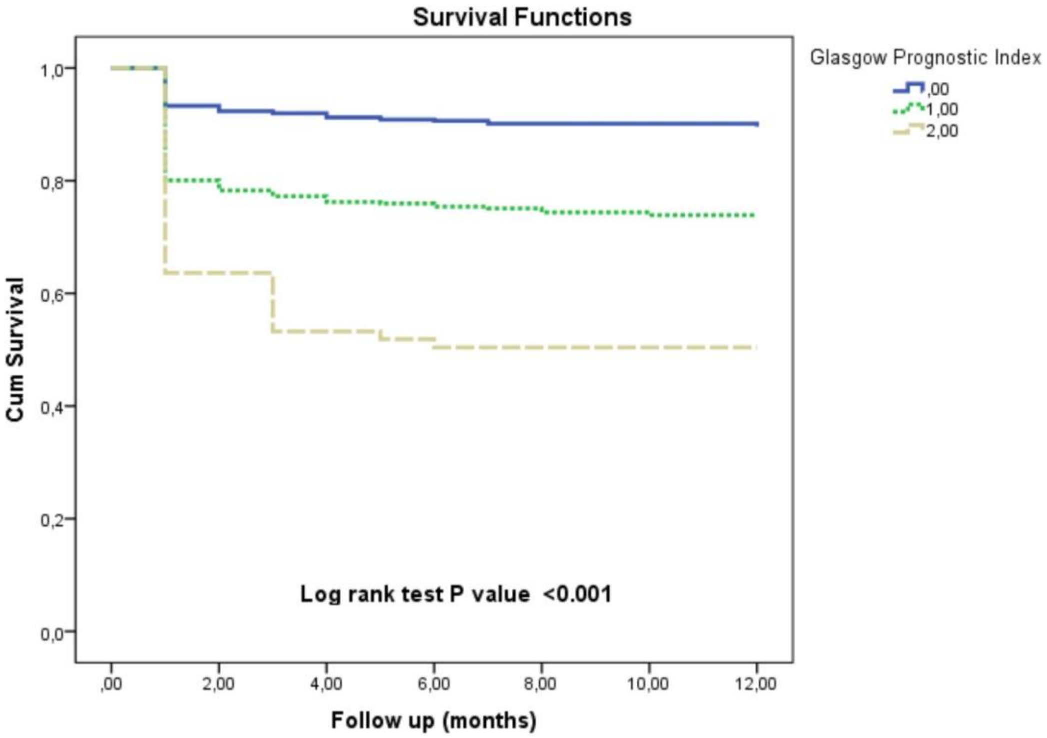

Figure S2. Kaplan-Meier survival analysis.

Table S1. Relationships between clinical characteristics and the GPS in patients followed in intensive care unit.

Baseline characteristics	GPS = 0 (*n* = 536)	GPS = 1 (*n* = 391)	GPS = 2 (*n* = 77)	P
Age, y	62.7 ± 13.6 [a,b]	67.9 ± 13.5 [a]	69.9 ± 12.9 [b]	<0.001
Age, median and min-max	63 (18–91) [a,b]	69 (20–94) [a]	72 (21–92) [b]	<0.001

Age, males	60.7 ± 13.0 ^{a,b}	65.3 ± 13.2^{a}	66.8 ± 13.6 ^{b}	<0.001
Age, females	67.5 ± 13.6 ^{a,b}	71.6 ± 13.0 ^{a,c}	76.5 ± 8.1 ^{b,c}	<0.001
Males	377 (70.3)^{a}	227 (58.1)^{a,c}	53 (68.8)^{c}	<0.001
Females	159 (29.7) ^{a}	164 (41.9) ^{a,c}	24 (31.2) ^{c}	<0.001
Body mass index, kg/cm^2	27.5 ± 3.9	27.4 ± 3.4	26.7 ± 2.3	0.641
Smoking	276 (51.5)^{a,b}	156 (39.9)^{a}	27 (35.1)^{b}	<0.001
Hypertension	328 (61.2)	261 (66.8)	55 (71.4)	0.084
Diabetes mellitus	158 (29.5)^{b}	142 (36.3)^{c}	35 (45.5)^{b,c}	0.006
Dyslipidemia	67 (12.5)	40 (10.2)	3 (3.9)	0.065
Coronary artery disease	189 (35.3)	146 (37.3)	29 (37.7)	0.781
Heart failure	66 (12.3)^{a,b}	83 (21.2)^{a}	15 (19.5)^{b}	0.001
Left ventricular ejection fraction, %	51.0 ± 10.9^{a}	47.7 ± 11.1^{a}	48.7 ± 10.8	<0.001
Cerebrovascular accident	32 (6.0)	35 (9.0)	9 (11.7)	0.087
Peripheral arterial disease	13 (2.4)	11 (2.8)	4 (5.2)	0.386
Chronic kidney disease	53 (9.9)^{a}	63 (16.1)^{a,c}	8 (10.4)^{c}	0.015
Chronic lung disease	34 (6.3)^{b}	41 (10.5)	10 (13.0)^{b}	0.027
Dementia	9 (1.7)	5 (1.3)	2 (2.6)	0.682
Atrial fibrillation	63 (11.8)^{a,b}	80 (20.5)^{a}	18 (23.4)^{b}	<0.001
Systolic blood pressure, mmHg	120.5 ± 19.0	121.3 ± 20.1	117.1 ± 20.3	0.254
Diastolic blood presssure, mmHg	74.1 ± 27.0	71.1 ± 11.9	70.6 ± 13.3	0.101
ICCU LOS	3.6 ± 4.5	5.3 ± 4.8	5.2 ± 5.4	<0.001
Total LOS	7.6 ± 7.3	10.9 ± 8.5	11.3 ± 9.4	<0.001
**Laboratory analysis**				
White blood cell count, 10^9/L	10.6 ± 3.9^{b}	11.0 ± 4.5^{c}	12.6 ± 5.7^{b,c}	0.001
Neutrophil count, 10^9/L	7.5 ± 4.5^{a,b}	8.2 ± 4.3^{a,c}	10.8 ± 6.0^{b,c}	<0.001
Hemoglobin, g/dL	13.6 ± 1.9^{a,b}	12.2 ± 2.2^{a,c}	11.1 ± 2.0^{b,c}	<0.001
Glucose, mg/dL	149.7 ± 73.8^{a}	176.9 ± 132.6^{a}	175 ± 92.7	0.025
Glomerular filtration rate, mL/min	90.3 ± 58.4^{a}	74.0 ± 34.4^{a}	76.6 ± 44.1	0.001
Creatinine, mg/dL	1.04 ± 0.79^{a}	1.26 ± 1.00^{a}	1.25 ± 0.76	<0.001
Triglycerides, mg/dL	125 (87–182)	107 (79–154)	125 (84–187)	0.141
Total cholesterol, mg/dL	182 (150–218)^{a,b}	164 (134–198)^{a,c}	136 (116–168)^{b,c}	<0.001
LDL cholesterol, mg/dL	118 (94–143)^{a,b}	104 (84–130)^{a,c}	88 (68–110)^{b,c}	<0.001
NT-pro-BNP, pg/mL	2069 (458–5808)	5600 (2415–11236)	5361 (1540–11670)	0.569
Total bilirubin, mg/dL	0.50 (0.3–0.85)	0.60 (0.40–1.00)	0.75 (0.45–1.20)	0.170
Alanine aminotransferase, IU/L	20 (15–30)^{b}	22 (13–33)	24 (16–46)^{b}	0.041
Aspartate aminotransferase, IU/L	32 (23–51)^{b}	34 (23–62)^{c}	46 (26–104)^{b,c}	0.012
**In-hospital medication/treatment**				
Aspirin	444 (82.8)	292 (74.7)	56 (72.7)	0.004
P2Y12 antagonists	399 (74.4)^{a,b}	252 (64.5)^{a}	46 (59.7)^{b}	0.001
Beta-blockers	382 (71.3)	260 (66.5)	49 (63.6)	0.179
CCB (dihydropyridine)	30 (5.6)	31 (7.9)	3 (3.9)	0.233
CCB (non-dihydropyridine)	11 (2.1)	10 (2.6)	0 (0.0)	0.357
ACEI or ARB	296 (55.2)	187 (47.8)	37 (48.1)	0.066
Oral anticoagulants	21 (3.9)	28 (7.2)	4 (5.2)	0.093
Heparin (all class)	454 (84.7)	312 (79.8)	62 (80.5)	0.136
Statins	380 (70.9)^{a,b}	235 (60.1)^{a}	46 (59.7)^{b}	0.001
Diuretics	85 (15.9)^{a}	104 (26.6)^{a}	17 (22.1)	<0.001
Spironolactone	37 (6.9)^{a,b}	54 (13.8)^{a}	13 (16.9)^{b}	<0.001
Nitrates	111 (20.7)	84 (21.5)	19 (24.7)	0.725
İnotropic agents	35 (6.5)^{a,b}	72 (18.4)^{a,c}	23 (29.9)^{b,c}	<0.001
Therapeutic hypothermia	5 (0.9)	9 (2.3)	1 (1.3)	0.235
Total parenteral nutrition	3 (0.6)^{a,b}	17 (4.4)^{a}	3 (3.9)^{b}	<0.001
Antimicrobial agents	100 (18.7)^{a,b}	131 (33.5)^{a,c}	46 (59.7)^{b,c}	<0.001

Abbreviations: GPS, Glasgow prognostic score; LDL, low density lipoprotein; NT-pro-BNP, N-terminal pro B-type natriuretic peptide; CCB, calcium channel blocker; ACEI, angiotensin converting enzyme inhibitor; ARB, angiotensin receptor blocker. Symbols (^{a,b,c}) indicate post-hoc analysis (Tukey or Bonferoni Corrected Mann-Whitney U Test).

Table S2. The distribution of diagnosis of ICCU patients on admission.

**Main Diagnosis**	**Total**	**GPS = 0 (*n* = 536)**	**GPS = 1 (*n* = 391)**	**GPS = 2 (*n* = 77)**	**P**
Acute coronary syndrome	656 (65.3%)	382(71.3%)	231(59.1 %)	43 (55.8 %)	0.001
Acute heart failure	130 (12.9 %)	40 (7.5 %)	74 (18.9 %)	16 (20.8 %)	0.001
Atrioventricular block,	45 (4.5 %)	25 (4.7 %)	18 (4.6%)	2 (2.6 %)	0.7
Ventricular arrhythmia	31 (3.1 %)	18 (3.4 %)	10 (2.6 %)	3 (3.9%)	0.06
Atrial fibrillation with rapid ventricular response	32 (3.2 %)	17 (3.2%)	12 (3.1 %)	3 (3.9 %)	0.9
Hypertensive emergency	19 (1.9 %)	6 (1.1 %)	11 (2.8 %)	2 (2.6 %)	0.1
Severely valvular disease	16 (1.6 %)	8 (1.5 %)	7 (1.8 %)	1 (1.3%)	0.9
Cardiopulmonary arrest	20 (2 %)	6 (1.1 %)	10 (2.6 %)	4(5.2%)	0.34
Pericardial tamponade	14 (1.4 %)	8 (1.5 %)	8 (2.0 %)	2 (2.6%)	0.07
Myocarditis	11 (1.1 %)	7 (1.3 %)	3 (0.8%)	1 (1.3%)	0.7
Others	28 (2.8 %)	18 (3.4 %)	9 (2.3%)	1 (1.3%)	0.4

Table S3. Relationships between clinical events and the GPS in patients followed in intensive care unit and followup period.

**Events**	**GPS = 0 (*n* = 536)**	**GPS = 1 (*n* = 391)**	**GPS = 2 (*n* = 77)**	**P**
Cardio-pulmonary arrest	24 (4.5)[a,b]	66 (16.9)[a,c]	20 (26.0)[b,c]	<0.001
Ventricular arrhythmia	17 (3.2)	19 (4.9)	5 (6.5)	0.237
Cerebrovascular accident	2 (0.4)	2 (0.5)	0 (0.0)	0.801
Acute kidney injury	26 (4.9)[a,b]	45 (11.5)a	7 (9.1)[b]	0.001
Death (In-hospital)	22 (4.1)[a,b]	64 (16.4)[a,c]	23 (29.9)[b,c]	<0.001
Death (Follow-up)	31 (5.8)[a,b]	36 (9.2)[a,c]	15 (19.5)[b,c]	<0.001
Rehospitalisation	83 (16.1)[a,b]	79 (24.3)[a,c]	17 (31.5)[b,c]	0.002
Reinfarct	19 (3.7)	12 (3.7)	2 (3.7)	1
Total death (12-months)	53 (9.9)[a,b]	100 (25.6)[a,c]	38 (49.4)[b,c]	<0.001
Total death, males (12-months)	34 (6.3) [a,b]	48 (12.2) [a,c]	28 (36.3) [b,c]	<0.001
Total death, females (12-months)	19 (3.6) [a,b]	52 (13.4) [a,c]	10 (16.1) [b,c]	<0.001
Total death <60 y (12-months)	9 (1.6) [b]	15 (3.8)	4 (5.1) [c]	<0.001
Total death ≥60 y (12-months)	44 (2.0) [a,b]	85 (9.6) [a,c]	34 (44.3) [b,c]	<0.001

Symbols (a,b,c) indicate post-hoc analysis.

Table S4. Logistic regression of in-hospital mortality for patients followed in intensive care unit.

	**Univariate analysis**			Multivariate analysis		
	**OR**	**95% CI**	**P**	OR	95% CI	P
GPS (1 vs. 0)	4.57	2.76–7.56	<0.001	4.52	1.20–16.91	0.025
GPS (2 vs. 0)	9.95	5.20–19.02	<0.001	7.01	1.39–35.15	0.018
Age	1.05	1.03–1.07	<0.001			
Hypertension	0.61	0.40–0.91	0.016			
Heart failure	1.82	1.13–2.91	0.013			
Chronic kidney disease	1.83	1.09–3.08	0.003			
Atrial fibrillation	2.21	1.39–3.50	0.001			
Hemoglobin	0.83	0.76–0.91	<0.001			
White blood cell count	1.09	1.05–1.13	<0.001			
Creatinine	1.43	1.21–1.70	<0.001			
Total cholesterol	0.98	0.98–0.99	<0.001			
NT-pro-BNP	1.06	1.02–1.08	<0.001	1.03	1.02–1.06	<0.001
Total bilirubin	1.30	1.01–1.69	0.042			
Alanine aminotransferase	1.00	1.00–1.00	<0.001			
Aspartate aminotransferase	1.00	1.00–1.00	<0.001			
Left ventricular ejection fraction	0.95	0.93–0.96	<0.001			
